# Biomolecular Events in Cancer Revealed by Attractor Metagenes

**DOI:** 10.1371/journal.pcbi.1002920

**Published:** 2013-02-21

**Authors:** Wei-Yi Cheng, Tai-Hsien Ou Yang, Dimitris Anastassiou

**Affiliations:** 1 Center for Computational Biology and Bioinformatics and Department of Electrical Engineering, Columbia University, New York, New York, United States of America; Jefferson Medical College/Thomas Jefferson University, United States of America

## Abstract

Mining gene expression profiles has proven valuable for identifying signatures serving as surrogates of cancer phenotypes. However, the similarities of such signatures across different cancer types have not been strong enough to conclude that they represent a universal biological mechanism shared among multiple cancer types. Here we present a computational method for generating signatures using an iterative process that converges to one of several precise attractors defining signatures representing biomolecular events, such as cell transdifferentiation or the presence of an amplicon. By analyzing rich gene expression datasets from different cancer types, we identified several such biomolecular events, some of which are universally present in all tested cancer types in nearly identical form. Although the method is unsupervised, we show that it often leads to attractors with strong phenotypic associations. We present several such multi-cancer attractors, focusing on three that are prominent and sharply defined in all cases: a mesenchymal transition attractor strongly associated with tumor stage, a mitotic chromosomal instability attractor strongly associated with tumor grade, and a lymphocyte-specific attractor.

## Author Summary

Cancer is known to be characterized by several unifying biological capabilities or “hallmarks.” However, attempts to computationally identify patterns, such as gene expression signatures, shared across many different cancer types have been largely unsuccessful. A typical approach has been to classify samples into mutually exclusive subtypes, each of which is characterized by a particular gene signature. Although occasional similarities of such signatures in different cancer types exist, these similarities have not been sufficiently strong to conclude that they reflect the same biological event. By contrast, we have developed a computational methodology that has identified some signatures of co-expressed genes exhibiting remarkable similarity across many different cancer types. These signatures appear as stable “attractors” of an iterative computational procedure that tends to collect mutually associated genes, so that its convergence can point to the core (“heart”) of the underlying biological co-expression mechanism. One of these “pan-cancer” attractors corresponds to a transdifferentiation of cancer cells empowering them with invasiveness and motility. Another represents a mitotic chromosomal instability of cancer cells. A third attractor is lymphocyte-specific.

## Introduction

Despite their type-specific features, cancers share some common traits, or “hallmarks,” related to, e.g., the abilities of some cancer cells to divide uncontrollably or to invade surrounding tissues [1]. Furthermore, it has been recognized that gene expression signatures resulting from analysis of cancer datasets can serve as surrogates of cancer phenotypes [2]. Therefore, it is reasonable to hypothesize that computational analysis of rich biomolecular cancer datasets may reveal signatures that are shared across many cancer types and are associated with specific cancer phenotypes. Such rich datasets become publicly available at an increasing rate from many sources, such as The Cancer Genome Atlas (TCGA). However, attempts to identify any such robust “bioinformatic hallmarks” of cancer have so far been largely unsuccessful.

Gene signatures may occasionally be found to exhibit similarities across multiple cancer types. However, to our knowledge no algorithm has ever produced a set of nearly identical signatures after independently and separately analyzing datasets from different cancer types.

There are various ways by which modules of co-expressed genes can be identified from rich datasets, some of which may be within the context of regulatory network discovery [3]. Clustering approaches can classify a selected set of genes into subsets each of which contains mutually related genes. Related techniques can also be used to classify samples into cancer subtypes [4]–[6], each characterized by a set of characteristic genes. One of the most powerful computational approaches for this task has been nonnegative matrix factorization (NMF) [7] combined with consensus clustering [8], resulting in successful subtype identification in several types of cancer.

The main objective addressed by techniques such as NMF is to reduce dimensionality by identifying a number of metagenes jointly representing the gene expression dataset as accurately as possible, in lieu of the whole set of individual genes. Each metagene in NMF is defined as a positive linear combination of the individual genes, so that its expression level is an accordingly weighted average of the expression levels of the individual genes. The identity of each resulting metagene is influenced by the presence of other metagenes within the objective of overall dimensionality reduction achieved by joint optimization.

By contrast, if the aim is exclusively to identify metagenes as surrogates of biomolecular events, then a fully unconstrained algorithm should be devised, without any effort to achieve dimensionality reduction, classification, mutual exclusivity, orthogonality, regulatory interaction inference, etc.

We can consider, for example, a hypothetical case in which we have found a cluster consisting of a number of co-expressed genes in a rich gene expression dataset. We may wish to scrutinize and “sharpen” this co-expression trying to identify the “heart” (core) of the genes that are most strongly co-expressed in that case. In the absence of a defining phenotype, we can continue applying an unsupervised methodology, as follows: We can first define a consensus metagene from the average expression levels of all genes in the cluster, and rank all the individual genes in terms of their association (defined numerically by some form of correlation) with that metagene. We can then replace the member genes of the cluster with an equal number of the top-ranked genes. Some of the original genes may naturally remain as members of the cluster, but some may be replaced, as this process will “attract” some other genes that are more strongly correlated with the cluster. We can now define a new metagene defined by the average expression levels of the genes in the newly defined cluster, and re-rank all the individual genes in terms of their association with that new metagene; and so on. It is intuitively reasonable to expect that this iterative process will eventually converge to a cluster that contains precisely the genes that are most associated with the metagene of the same cluster, so that any other individual genes will be less strongly associated with the metagene. We can think of this particular cluster defined by the convergence of this iterative process as an “attractor,” i.e., a module of co-expressed genes to which many other gene sets with close but not identical membership will converge using the same computational methodology.

The above description represents a simplified conceptual introduction of the computational methodology presented in this paper. Rather than using the average of the expression values in gene clusters of a particular size, the “attractors” are metagenes defined as weighted averages of all genes where each individual gene has a nonnegative weight, just like the metagenes defined using NMF [7]. We found that, given a rich (loosely defined as containing at least 200 samples) dataset represented by a gene expression matrix, such metagenes can be naturally identified as stable and precise attractors using a simple iterative approach. We experimentally verified these nice convergence properties without any exception after trying numerous times the method described in this paper on such rich datasets.

This methodology is totally unsupervised, as it does not make use of any phenotypic association. As we show in this paper, however, once identified, a metagene attractor is likely to be found associated with a phenotype.

We found that several attractor metagenes are present in nearly identical form in multiple cancer types. This provides an additional opportunity to combine the powers of a large number of rich datasets to focus, at an even sharper level, on the core genes of the underlying mechanism. For example, this methodology can precisely point to the causal (driver) oncogenes within amplicons to be among very few candidate genes. Importantly, this can be done from rich gene expression data, which already exist in abundance, without making any use of sequencing data.

We identified several attractors, each of which has the potential to lead to corresponding testable biological hypotheses after scrutinizing their top-ranked genes and finding a putative underlying mechanism. For the purposes of this paper we present the general methodology for the benefit of the research community together with a listing of the attractors in six datasets from three cancer types (ovarian, colon, breast).

Here, we focus on a few interesting cancer-associated attractors that we found present in multiple cancer types. Particular emphasis is given to what we consider to be three key “bioinformatic hallmarks” of cancer, related to the ability of cancer cells to invade surrounding tissues; to divide uncontrollably; and the ability of the organism to recruit the immune system to fight cancer: a tumor stage-associated mesenchymal transition attractor, a tumor grade-associated mitotic chromosomal instability (mitotic CIN) attractor, and a lymphocyte-specific attractor.

## Results

### Derivation of Attractor Metagenes

Given a nonnegative measure 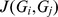 of pairwise association between genes 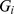 and 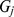, we define an attractor metagene 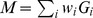 to be a linear combination of the individual genes with weights 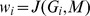. The association measure 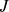 is assumed to have minimum possible value 0 and maximum possible value 1, so the same is true for the weights. It is also assumed to be scale-invariant, therefore it is not necessary for the weights to be normalized so that they add to 1, and the metagenes can still be thought of as expressing a normalized weighted average of the expression levels of the individual genes. See Materials and Methods for the choice of the measure 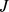.

According to this definition, the genes with the highest weights in an attractor metagene will have the highest association with the metagene (and, by implication, they will tend to be highly associated among themselves) and so they will often represent a biomolecular event reflected by the co-expression of these top genes. This can happen, e.g., when a biological mechanism is activated, or when a copy number variation (CNV), such as an amplicon, is present, in some of the samples included in the expression matrix. In the following we use the term “attractor” for simplicity to refer to an attractor metagene, and the term “top genes” to refer to the genes with the highest weights in the attractor. The definition of an attractor metagene can readily be generalized to include features other than gene expression, such as methylation values. It can also be used in datasets of any objects (not necessarily genes) characterized by any type of feature vectors, with applications in other disciplines, such as social and economic sciences.

The computational problem of identifying attractor metagenes given an expression matrix can be addressed heuristically using a simple iterative process: Starting from a particular seed (or “attractee”) metagene 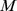, a new metagene is defined in which the new weights are 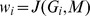. The same process is then repeated in the next iteration resulting in a new set of weights, and so forth. In all gene expression datasets that we tried we found that this process converges to a limited number of stable attractors. Each attractor is defined by a precise set of weights, which are reached with high accuracy typically within 10 or 20 iterations.

This algorithmic behavior with nice convergence properties is not surprising, because if a metagene represents co-expressed genes, then the next iteration will naturally “attract” other similarly co-expressed genes, and so forth, until there are no other genes more associated with the top genes than those genes themselves. Furthermore, the set of the few genes with the highest weight are likely to represent the “heart” (core) of the underlying biomolecular event. In support of this concept, the association of any of the top-ranked individual genes with the attractor metagene is consistently and significantly higher than the pairwise association between any of these genes, suggesting that the set of these top genes jointly comprise a proxy representing a biomolecular event better than each of the individual genes would.

Indeed, related versions of the signatures identified by attractors in this paper have been previously identified in various contexts in individual cancer types, often intermingled with additional genes. However, the contribution of our work is that these signatures are found as pan-cancer biomolecular events, sharply pointing to the underlying mechanism. Therefore the top genes of the attractors will be appropriate for being used as biomarkers or for understanding the underlying biology. For example, one of the attractors that we identified (the “mitotic chromosomal instability” attractor, described below) has previously been found in approximate forms among sets of genes described generally [9] as “proliferation” or “cell cycle related” markers, while the actual attractor points much more sharply to particular elements in the structure of the kinetochore-microtubule interface.

A reasonable implementation of an “exhaustive” search of attractor metagenes is to start from each individual gene as a seed (“attractee”) assigning a weight of 1 to that gene, and 0 to all the other genes. Each gene participating in a particular co-expression event will then lead to the same attractor when used as attractee. The computational implementation of the algorithm is described in Materials and Methods. We note that a dual method can be used to identify attractor “metasamples” as representatives of subtypes, and we can also combine such metasamples with the attractor metagenes in various ways to achieve biclustering, but this topic is not examined in this paper.

We analyzed six datasets, two from ovarian cancer, two from breast cancer and two from colon cancer (Supplementary Text S1). In each case, we identified general (Supplementary Table S1) and genomically localized (Supplementary Table S2) attractors and we found that many among them appear in similar forms in all six datasets using particular merging and ranking criteria in each case (Materials and Methods and Supplementary Text S1). Following are descriptions of some of our results, starting with the three strongest multi-cancer attractors.

### Mesenchymal Transition Attractor Metagene

This attractor contains mostly epithelial-mesenchymal transition (EMT)-associated genes. Table 1 provides a listing of the top 100 genes based on their average mutual information (Materials and Methods) with their corresponding attractor metagenes.

**Table 1:** Top 100 genes of the mesenchymal transition attractor based on six datasets.

Rank	Gene Symbol	Avg MI	Rank	Gene Symbol	Avg MI
1	COL5A2	0.814	51	SULF1	0.505
2	VCAN	0.775	52	LOXL1	0.502
3	SPARC	0.766	53	PRRX1	0.502
4	THBS2	0.758	54	PPAPDC1A	0.499
5	FBN1	0.749	55	COL10A1	0.498
6	COL1A2	0.749	56	ITGA11	0.495
7	COL5A1	0.747	57	NTM	0.494
8	FAP	0.734	58	MXRA8	0.494
9	AEBP1	0.711	59	FIBIN	0.493
10	CTSK	0.709	60	WISP1	0.483
11	COL3A1	0.688	61	RCN3	0.483
12	COL1A1	0.683	62	TNFAIP6	0.481
13	SERPINF1	0.674	63	ECM2	0.480
14	COL6A3	0.669	64	HTRA1	0.480
15	CDH11	0.663	65	EFEMP2	0.478
16	GLT8D2	0.658	66	MXRA5	0.474
17	LUM	0.654	67	ACTA2	0.472
18	MMP2	0.654	68	LOX	0.470
19	DCN	0.650	69	ITGBL1	0.466
20	CCDC80	0.637	70	PMP22	0.465
21	POSTN	0.631	71	P4HA3	0.464
22	CTHRC1	0.616	72	PTRF	0.463
23	ADAM12	0.613	73	CALD1	0.460
24	COL6A2	0.608	74	HEG1	0.458
25	MSRB3	0.608	75	NEXN	0.455
26	OLFML2B	0.607	76	NID2	0.455
27	INHBA	0.600	77	TAGLN	0.455
28	FSTL1	0.600	78	FAM26E	0.452
29	SFRP2	0.596	79	ZNF521	0.452
30	SNAI2	0.577	80	SFRP4	0.451
31	CRISPLD2	0.574	81	PALLD	0.450
32	PCOLCE	0.571	82	OLFML1	0.447
33	PDGFRB	0.567	83	FILIP1L	0.447
34	BGN	0.565	84	TIMP3	0.445
35	COL12A1	0.560	85	SPON2	0.443
36	ANGPTL2	0.555	86	SPOCK1	0.443
37	COPZ2	0.553	87	COL8A2	0.441
38	CMTM3	0.549	88	GPC6	0.438
39	ASPN	0.547	89	PDPN	0.437
40	FN1	0.545	90	GFPT2	0.436
41	CNRIP1	0.540	91	LHFP	0.436
42	FNDC1	0.538	92	GREM1	0.436
43	LRRC15	0.533	93	TGFB1I1	0.435
44	COL11A1	0.529	94	C1S	0.433
45	ANTXR1	0.528	95	EDNRA	0.432
46	RAB31	0.527	96	GAS1	0.431
47	FRMD6	0.524	97	NOX4	0.431
48	TSHZ3	0.520	98	FBLN2	0.428
49	THY1	0.519	99	TCF4	0.428
50	NNMT	0.519	100	NUAK1	0.427

The consistency of the attractor is established by the fact (Supplementary Table S1) that there are many genes (COL5A2, COL1A2, SPARC, CTSK, FBN1, VCAN, AEBP1, SERPINF1) that are among the top 50 genes in the attractors of *all* six datasets. The corresponding *P* value is less than 10^−7^ by permutation test (Materials and Methods). Similar results are found in other solid cancer types in all cases that we tried.

This is a stage-associated attractor, in which the signature is significantly present only when a particular level of invasive stage, specific to each cancer type, has been reached. Supplementary Table S3 demonstrates this phenomenon in three cancer datasets from different types (breast, ovarian and colon) that were annotated with clinical staging information, by providing a listing of differentially expressed genes, ranked by fold change, when ductal carcinoma in situ (DCIS) progresses to invasive ductal carcinoma; ovarian cancer progresses to stage III; and colon cancer progresses to stage II. In all three cases, the attractor is highly enriched among the top genes. Specifically, among the top 100 differentially expressed genes, the number of attractor genes included in Table 1 is 47 in breast cancer, 42 in ovarian cancer and 37 in colon cancer. The corresponding *P* values are 2×10^−93^, 4×10^−80^ and 8×10^−78^, respectively.

This attractor has been previously identified with remarkable accuracy as representing a particular kind of mesenchymal transition of cancer cells present in all types of solid cancers tested leading to a published list of top 64 genes [10], [11]. This list was generated using a supervised algorithm using association with tumor stage. Indeed 52 of these top 64 genes also appear in Table 1 (*P*<10^−114^), and furthermore all top 19 genes of Table 1 are among the 64. We found that most of the genes of the signature were expressed by the cancer cells themselves, and not by the surrounding stroma, at least in a neuroblastoma xenograft model that we tried [11]. We also found that the signature is associated with prolonged time to recurrence in glioblastoma [12]. Related versions of the same signature were previously found to be associated with resistance to neoadjuvant therapy in breast cancer [13]. These results are consistent with the finding that EMT induces cancer cells to acquire stem cell properties [14]. It has been hypothesized that EMT is a key mechanism for cancer cell invasiveness and motility [15]–[17]. The attractor, however, appears to represent a more general phenomenon of transdifferentiation present even in nonepithelial cancers such as neuroblastoma, glioblastoma and Ewing's sarcoma.

Although similar signatures are often labeled as “stromal,” because they contain many stromal markers such as α-SMA and fibroblast activation protein, the fact that most of the genes of the signature were expressed by xenografted cancer cells [11], and not by mouse stromal cells, suggests that this particular attractor of coordinately expressed genes represents cancer cells having undergone a mesenchymal transition. The signature may indicate a non-fibroblastic transition, as occurs in glioblastoma, in which case collagen *COL11A1* is not co-expressed with the other genes of the attractor. We have hypothesized that a full fibroblastic transition of the cancer cells occurs when cancer cells encounter adipocytes [11], in which case they may well assume the duties of cancer-associated fibroblasts (CAFs) in some tumors [1]. In that case, the best proxy of the signature [10] is *COL11A1* and the strongly co-expressed genes *THBS2* and *INHBA*. Indeed, the 64 genes of the previously identified signature were found from multi-cancer analysis [10] as the genes whose expression is consistently most associated with that of *COL11A1*.

The only EMT-inducing transcription factor found upregulated in the xenograft model [11] is SNAI2 (Slug), and it is also the one most associated with the signature in publicly available datasets. We also found that the microRNAs most highly associated with this attractor are miR-214, miR-199a, and miR-199b. Interestingly, miR-214 and miR-199a were found to be jointly regulated by another EMT-inducing transcription factor, TWIST1 [18].

**Figure 1:**
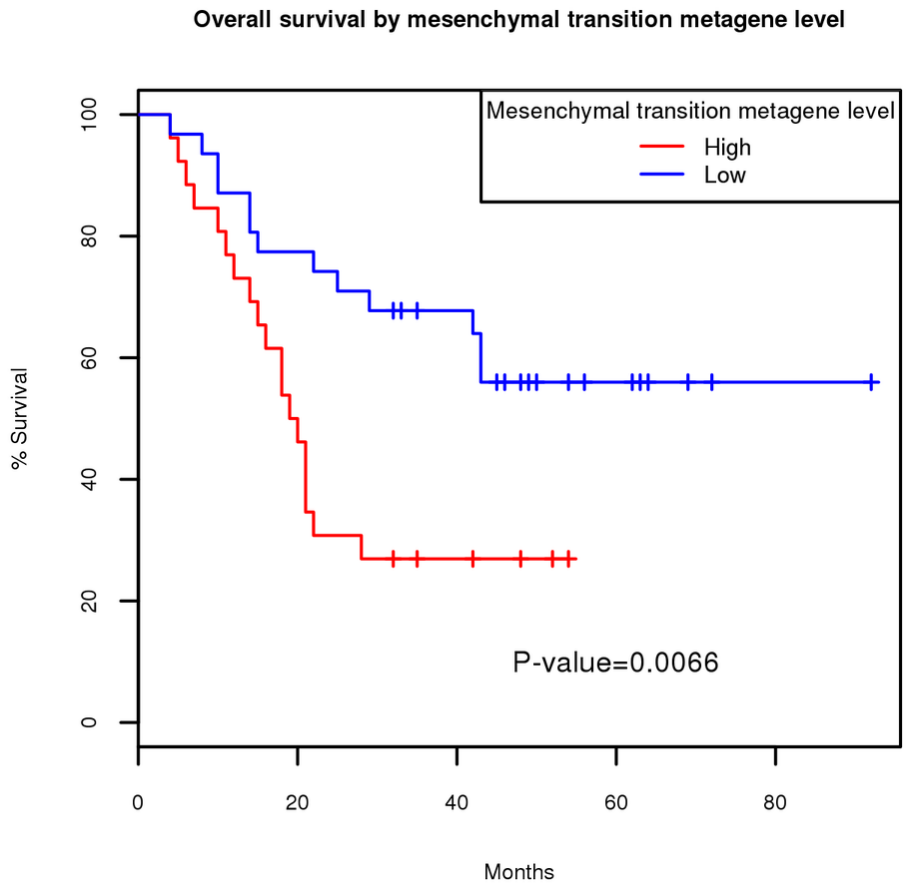
Kaplan-Meier curves of mesenchymal transition attractor metagene in oral squamous cell carcinoma dataset. Gene expression data from 57 patients (GSE25104) were divided into two groups: high mesenchymal transition metagene level and low mesenchymal transition metagene level depending on whether the metagene expression value exceeding the mean of the 57 patients. The *P* value of the association was determined by log-rank test.

The expression of the mesenchymal transition attractor indicates that the tumor is actively invasive at the specific sample site, so its prognostic value is cancer type and stage specific. As an example, we analyzed an oral squamous cell carcinoma dataset deposited in the Gene Expression Omnibus (GEO) under accession number GSE25104. The corresponding Kaplan-Meier survival curve (*P* = 0.0066) is shown in Figure 1.

### Mitotic CIN Attractor Metagene

This attractor contains mostly kinetochore-associated genes. Table 2 provides a listing of the top 100 genes based on their average mutual information (Materials and Methods) with their corresponding attractor metagenes, starting from *CENPA*, which encodes for a histone H3-like centromeric protein.

**Table 2:** Top 100 genes of the mitotic CIN attractor based on six datasets.

Rank	Gene Symbol	Avg MI	Rank	Gene Symbol	Avg MI
1	CENPA	0.720	51	CDCA8	0.532
2	DLGAP5	0.693	52	CDC45	0.528
3	MELK	0.684	53	KIF18A	0.524
4	BUB1	0.674	54	HMMR	0.506
5	KIF2C	0.660	55	TOP2A	0.505
6	KIF20A	0.658	56	CENPF	0.503
7	KIF4A	0.656	57	ZWINT	0.503
8	CCNA2	0.654	58	PLK1	0.501
9	CCNB2	0.652	59	RAD51AP1	0.501
10	NCAPG	0.649	60	FAM83D	0.498
11	TTK	0.642	61	E2F8	0.497
12	CEP55	0.638	62	CENPE	0.497
13	CCNB1	0.632	63	MKI67	0.492
14	CDK1	0.629	64	CENPN	0.491
15	HJURP	0.626	65	MAD2L1	0.489
16	CDC20	0.624	66	CHEK1	0.486
17	CDCA5	0.615	67	GTSE1	0.477
18	NCAPH	0.615	68	RAD51	0.475
19	BUB1B	0.609	69	SGOL2	0.474
20	KIF23	0.592	70	PARPBP	0.469
21	KIF11	0.591	71	TRIP13	0.467
22	BIRC5	0.589	72	SHCBP1	0.465
23	NUF2	0.587	73	DTL	0.465
24	TPX2	0.586	74	CENPL	0.462
25	AURKB	0.582	75	FEN1	0.461
26	RACGAP1	0.580	76	FANCI	0.461
27	NUSAP1	0.580	77	FBXO5	0.459
28	ASPM	0.579	78	ECT2	0.457
29	MCM10	0.579	79	MND1	0.456
30	PRC1	0.576	80	CDC25C	0.456
31	DEPDC1B	0.572	81	PBK	0.456
32	UBE2C	0.569	82	KPNA2	0.452
33	UBE2T	0.567	83	RAD54L	0.452
34	NEK2	0.566	84	ESPL1	0.447
35	FOXM1	0.565	85	CDCA2	0.446
36	NDC80	0.556	86	FAM64A	0.440
37	CDCA3	0.556	87	CENPK	0.436
38	FAM54A	0.553	88	MYBL2	0.435
39	ANLN	0.551	89	SPAG5	0.434
40	KIF15	0.548	90	EZH2	0.431
41	STIL	0.547	91	SMC4	0.430
42	EXO1	0.542	92	TACC3	0.428
43	AURKA	0.540	93	C11orf82	0.427
44	PTTG1	0.539	94	MASTL	0.426
45	OIP5	0.539	95	ASF1B	0.426
46	RRM2	0.539	96	PTTG3P	0.425
47	DEPDC1	0.539	97	CENPW	0.424
48	CDKN3	0.538	98	ORC1	0.424
49	KIF14	0.537	99	HELLS	0.422
50	SPC25	0.534	100	TK1	0.421

The consistency of the attractor is established by the fact (Supplementary Table S1) that there are many genes (CENPA, DLGAP5, KIF2C, CCNB2, MELK, CCNA2, KIF20A, HJURP, NUSAP1, BUB1, TTK, KIF11, NCAPH) that are among the top 50 genes in the attractors of all six datasets. The corresponding *P* value is less than 10^−7^ by permutation test (Materials and Methods). Similar results are found in other solid cancer types in all cases that we tried.

Contrary to the stage-associated mesenchymal transition attractor, this is a grade-associated attractor, in which the signature is significantly present only when an intermediate level of tumor grade is reached. Supplementary Table S4 demonstrates this phenomenon in three cancer datasets from different types (breast, ovarian and bladder) that were annotated with tumor grade information, by providing a listing of differentially expressed genes, ranked by fold change, when grade G2 is reached. In all three cases, the attractor is highly enriched among the top genes. Specifically, among the top 100 differentially expressed genes, the number of attractor genes included Table 2 is 40 in breast cancer, 38 in ovarian cancer and 27 in colon cancer. The corresponding *P* values are 4×10^−74^, 3×10^−69^ and 3×10^−49^, respectively. Consistently, a similar “gene expression grade index” signature [19] was previously found differentially expressed between histologic grade 3 and histologic grade 1 breast cancer samples. Furthermore, that same signature [19] was found capable of reclassifying patients with histologic grade 2 tumors into two groups with high versus low risks of recurrence.

This attractor is associated with chromosomal instability (CIN), as evidenced from the fact that another similar gene set comprising a “signature of chromosomal instability” [20] was previously derived from multiple cancer datasets purely by identifying the genes that are most correlated with a measure of aneuploidy in tumor samples. This led to a 70-gene signature referred to as “CIN70.” Indeed 31 of these 70 genes appear in Table 2 (*P*<10^−53^). However, several top genes of the attractor, such as *CENPA*, *DLGAP5*, *KIF2C*, *BUB1* and *CCNA2* are not present in the CIN70 list. Mitotic CIN is increasingly recognized [21] as a widespread multi-cancer phenomenon.

The attractor is characterized by overexpression of kinetochore-associated genes, which is known [22] to induce CIN. Overexpression of several of the genes of the attractor, such as the top gene *CENPA* [23], as well as *MAD2L1* [24] and *TPX2* [25], has also been independently previously found associated with CIN. Included in the mitotic CIN attractor are key components of mitotic checkpoint signaling [26], such as *BUB1B*, *MAD2L1* (aka *MAD2*), *CDC20*, and *TTK* (aka *MSP1*). Also among the genes in the attractor is *MKI67* (aka *Ki-67*), which has been widely used as a proliferation rate marker in cancer.

Among transcription factors, we found *MYBL2* (aka *B-Myb*) and *FOXM1* to be strongly associated with the attractor. They are already known to be sequentially recruited to promote late cell cycle gene expression [27] to prepare for mitosis.

Inactivation of the retinoblastoma (RB) tumor suppressor promotes CIN [28] and the expression of the attractor signature. Indeed, a similar expression of a “proliferation gene cluster [29]” was found strongly associated with the human papillomavirus E7 oncogene, which abrogates RB protein function and activates E2F-regulated genes. Consistently, many among the genes of the attractor correspond to E2F pathway genes controlling cell division or proliferation. Among the E2F transcription factors, we found that E2F8 and E2F7 are most strongly associated with the attractor.

### Lymphocyte-Specific Attractor Metagene

This attractor consists mainly of lymphocyte-specific genes with prominent presence of *CD53*, *PTPRC*, *LAPTM5*, *DOCK2*, *LCP2* and *IL10RA*. It is strongly associated with the expression of microRNA miR-142 as well as with particular hypermethylated and hypomethylated gene signatures [30]. There is also significant overlap between the sets of hypomethylated and overexpressed genes, suggesting that their expression is triggered by hypomethylation. Gene set enrichment analysis reveals that the attractor is found enriched in genes known to be preferentially expressed in differentiation into lymphocytes [31]. Table 3 provides a listing of the top 100 genes of the lymphocyte-specific attractor based on their average mutual information (Materials and Methods) with their corresponding attractor metagenes.

**Table 3:** Top 100 genes of the lymphocyte-specific attractor based on six datasets.

Rank	Gene Symbol	Avg MI	Rank	Gene Symbol	Avg MI
1	PTPRC	0.782	51	NCF1	0.560
2	CD53	0.768	52	CCL5	0.557
3	LCP2	0.739	53	LST1	0.557
4	LAPTM5	0.708	54	CD3D	0.553
5	DOCK2	0.699	55	RCSD1	0.548
6	IL10RA	0.699	56	FGL2	0.538
7	CYBB	0.698	57	HCST	0.538
8	CD48	0.691	58	MARCH1	0.538
9	ITGB2	0.679	59	FERMT3	0.536
10	EVI2B	0.675	60	FCGR2B	0.533
11	MS4A6A	0.673	61	GIMAP5	0.530
12	TFEC	0.659	62	MYO1F	0.530
13	SLA	0.657	63	KLHL6	0.530
14	TNFSF13B	0.657	64	GIMAP1	0.527
15	C1orf162	0.656	65	CD163	0.524
16	SAMSN1	0.652	66	CLEC7A	0.522
17	PLEK	0.649	67	CCR1	0.518
18	GMFG	0.647	68	GBP5	0.517
19	GIMAP4	0.647	69	NCF2	0.516
20	SASH3	0.645	70	HLA-DPA1	0.516
21	EVI2A	0.638	71	RNASE6	0.515
22	SRGN	0.638	72	CD14	0.515
23	AIF1	0.636	73	FAM26F	0.511
24	LAIR1	0.627	74	CD4	0.510
25	FYB	0.625	75	FCGR1A	0.506
26	FCER1G	0.623	76	GZMA	0.506
27	MPEG1	0.621	77	GPR183	0.505
28	CD86	0.621	78	CD84	0.505
29	C3AR1	0.611	79	NKG7	0.504
30	C1QB	0.608	80	C1QA	0.502
31	CD2	0.606	81	CD300LF	0.500
32	HCLS1	0.599	82	FPR3	0.499
33	HCK	0.592	83	PARVG	0.496
34	MNDA	0.587	84	TRAF3IP3	0.494
35	CD37	0.587	85	TYROBP	0.492
36	LY96	0.585	86	LPXN	0.492
37	CCR5	0.585	87	GIMAP8	0.492
38	ARHGAP9	0.580	88	MS4A7	0.490
39	CD52	0.580	89	IL2RB	0.489
40	GPR65	0.580	90	CD300A	0.488
41	GIMAP6	0.578	91	IGSF6	0.488
42	SLAMF8	0.577	92	SELPLG	0.488
43	WIPF1	0.577	93	FCGR2A	0.487
44	MS4A4A	0.574	94	NCKAP1L	0.483
45	ARHGAP15	0.573	95	DOK2	0.483
46	HAVCR2	0.567	96	CD247	0.481
47	ARHGAP30	0.566	97	SELL	0.480
48	CLEC4A	0.566	98	GZMK	0.479
49	TAGAP	0.564	99	CCR2	0.479
50	CYTIP	0.563	100	LY86	0.479

The gene membership of the attractor provides hints about the underlying immune mechanism, which could be valuable towards generating hypotheses for potential immunotherapies such as adoptive transfer of lymphocytes. For example, the presence of the signal-transducing *LCP2* (aka *SLP-76*) gene, together with the adaptor *FYB* (aka *ADAP*), suggests the formation of the SLP-76-ADAP adaptor module, which is known to regulate lymphocyte co-stimulation mediated by integrin ITGB2 (aka LFA-1), another prominent gene in the attractor [32].

### Association of the Three Main Attractor Metagenes with Prognosis in Breast Cancer

We found that each of the above three main attractors under particular conditions is highly prognostic in breast cancer by analysing the METABRIC discovery breast cancer dataset [33] which includes both expression as well as survival data.

#### Mesenchymal transition attractor

**Figure 2:**
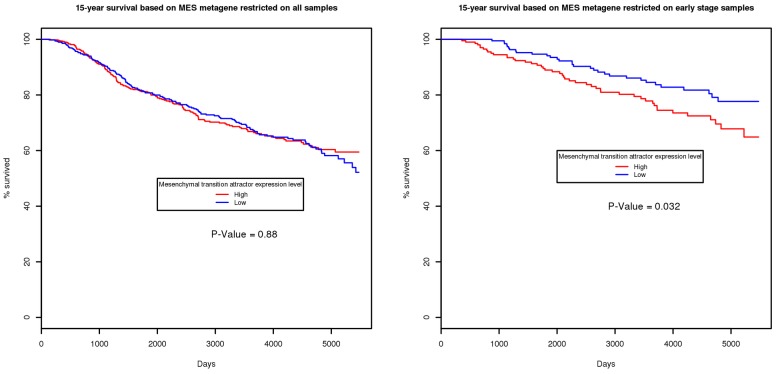
Kaplan-Meier curve of mesenchymal transition attractor metagenes in breast cancer dataset. The mesenchymal transition attractor metagene is most prominent in the early stage of breast cancer. The survival curve of the full dataset is insignificant (left). However, when the samples are restricted to only those at early stage (with no positive lymph nodes and tumor size less than 30 mm), the association between the mesenchymal transition attractor and the survival becomes significant (right), with *P* = 0.032.

In breast cancer, the mesenchymal transition attractor is expressed very early, as cancer becomes invasive. The presence of the attractor in a particular sample of high-stage tumor in not as informative, because of heterogeneity. On the other hand we found that the presence of the attractor in early-stage tumors is highly prognostic, consistent with the hypothesis that it indicates increased invasiveness. As shown in Figure 2, the Kaplan-Meier 15-year survival curves of the mesenchymal transition attractor using all samples does not show any significance. However, when we restrict the samples to early stage patients, defined as having no positive lymph nodes and tumor size less than 30 mm, the association between the attractor and survival became significant (*P* = 0.032).

#### Mitotic CIN attractor

**Figure 3:**
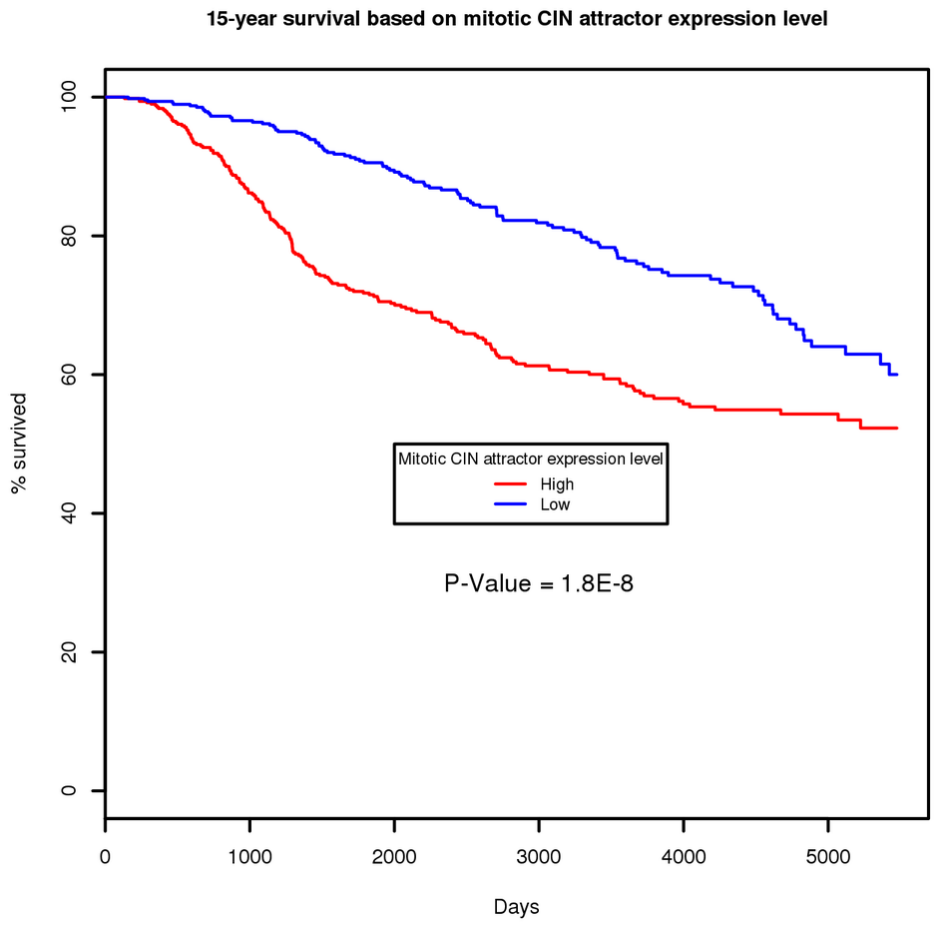
Kaplan-Meier curve of mitotic CIN attractor metagene in breast cancer dataset. To evaluate the association between the mitotic CIN metagene expression and the 15-year survival, patients were divided into two groups: high mitotic CIN and low mitotic CIN. This binary expression level was determined by whether the mitotic CIN metagene expression value exceeding the mean of the patients. The *P* value of the association based on log-rank test is 1.8×10^−8^.

The expression of the mitotic CIN attractor indicates that the tumor is dividing uncontrollably and therefore, in all cases, the attractor is highly prognostic for survival. The corresponding Kaplan-Meier 15-year survival curve (*P*<2×10^−8^) is shown in Figure 3. Furthermore, we ranked all the genes in terms of the concordance index [34] between the survival and the individual gene's expression value from the same rich dataset. Table 4 shows the top 100 genes, within which 47 (indicated by underline and boldface), including the top six, are also among the genes shown in Table 2 (*P* = 2×10^−98^ by Fisher's exact test). This extraordinary enrichment (also note that eight among the top ten genes, including the top three, are among the genes of Table 2) demonstrates that the members of the mitotic CIN attractor are, among all genes, the most prognostic ones, at least in breast cancer.

**Table 4:** List of top-ranked genes in terms of survival concordance index of the METABRIC discovery dataset demonstrating enrichment of the mitotic CIN attractor.

Rank	Gene Symbol	Concordance Index	Rank	Gene Symbol	Concordance Index
1	**CDCA5**	0.670	51	PRR11	0.639
2	**AURKA**	0.663	52	LOC651816	0.638
3	**KIF20A**	0.662	53	KRT80	0.638
4	TROAP	0.661	54	C15orf42	0.637
5	**UBE2C**	0.659	55	SGOL1	0.637
6	**AURKA**	0.658	56	GPI	0.637
7	**FAM83D**	0.657	57	**CEP55**	0.637
8	SHMT2	0.655	58	**MCM10**	0.636
9	**UBE2C**	0.655	59	PKMYT1	0.635
10	**CCNB2**	0.653	60	**CENPN**	0.635
11	**TPX2**	0.653	61	C20orf24	0.635
12	**EXO1**	0.653	62	SPC24	0.635
13	ORC6	0.653	63	RIPK4	0.635
14	**CENPA**	0.653	64	TOMM40	0.634
15	C1orf106	0.652	65	**ANLN**	0.634
16	**GTSE1**	0.652	66	ADRM1	0.634
17	**MELK**	0.651	67	**NCAPG**	0.633
18	STIP1	0.651	68	**CDCA8**	0.633
19	**SPC25**	0.649	69	AIF1L	0.633
20	**CENPA**	0.649	70	MRPS5	0.633
21	GARS	0.649	71	GPR56	0.633
22	**MELK**	0.649	72	PEX13	0.633
23	UCK2	0.648	73	ENO1	0.633
24	**HJURP**	0.648	74	NUTF2	0.633
25	**PTTG1**	0.647	75	MEMO1	0.632
26	CBX2	0.646	76	TXNRD1	0.632
27	CCNE1	0.646	77	SLC7A5	0.631
28	**PLK1**	0.646	78	**FOXM1**	0.631
29	**KIF2C**	0.645	79	**KIF14**	0.631
30	**CCNA2**	0.645	80	PPP1R14B	0.631
31	GMPSP1	0.645	81	**FAM54A**	0.630
32	**AURKB**	0.645	82	C20orf24	0.630
33	**BUB1**	0.644	83	SGOL1	0.630
34	**TRIP13**	0.643	84	NUP93	0.630
35	**FOXM1**	0.643	85	ZNF695	0.630
36	**CDC20**	0.643	86	**BIRC5**	0.630
37	LOC731049	0.642	87	**CENPL**	0.630
38	POLQ	0.642	88	SOX11	0.630
39	GSK3B	0.642	89	**KIF23**	0.629
40	CCNE1	0.642	90	SLC52A2	0.629
41	**KIF4A**	0.641	91	AIF1L	0.629
42	**PRC1**	0.641	92	**FEN1**	0.629
43	LAD1	0.641	93	CDC25A	0.629
44	**FAM64A**	0.641	94	**CDCA3**	0.628
45	SAPCD2	0.641	95	TMEM132A	0.628
46	**RACGAP1**	0.641	96	**CENPE**	0.628
47	POLR2D	0.641	97	NACC2	0.628
48	CKAP2L	0.640	98	**TTK**	0.628
49	**PTTG1**	0.640	99	SNRPA1	0.628
50	ECE2	0.639	100	MMP15	0.628

The 47 underlined genes are also among the top 100 genes of the mitotic CIN attractor (Table 2).

#### Lymphocyte-specific attractor

**Figure 4:**
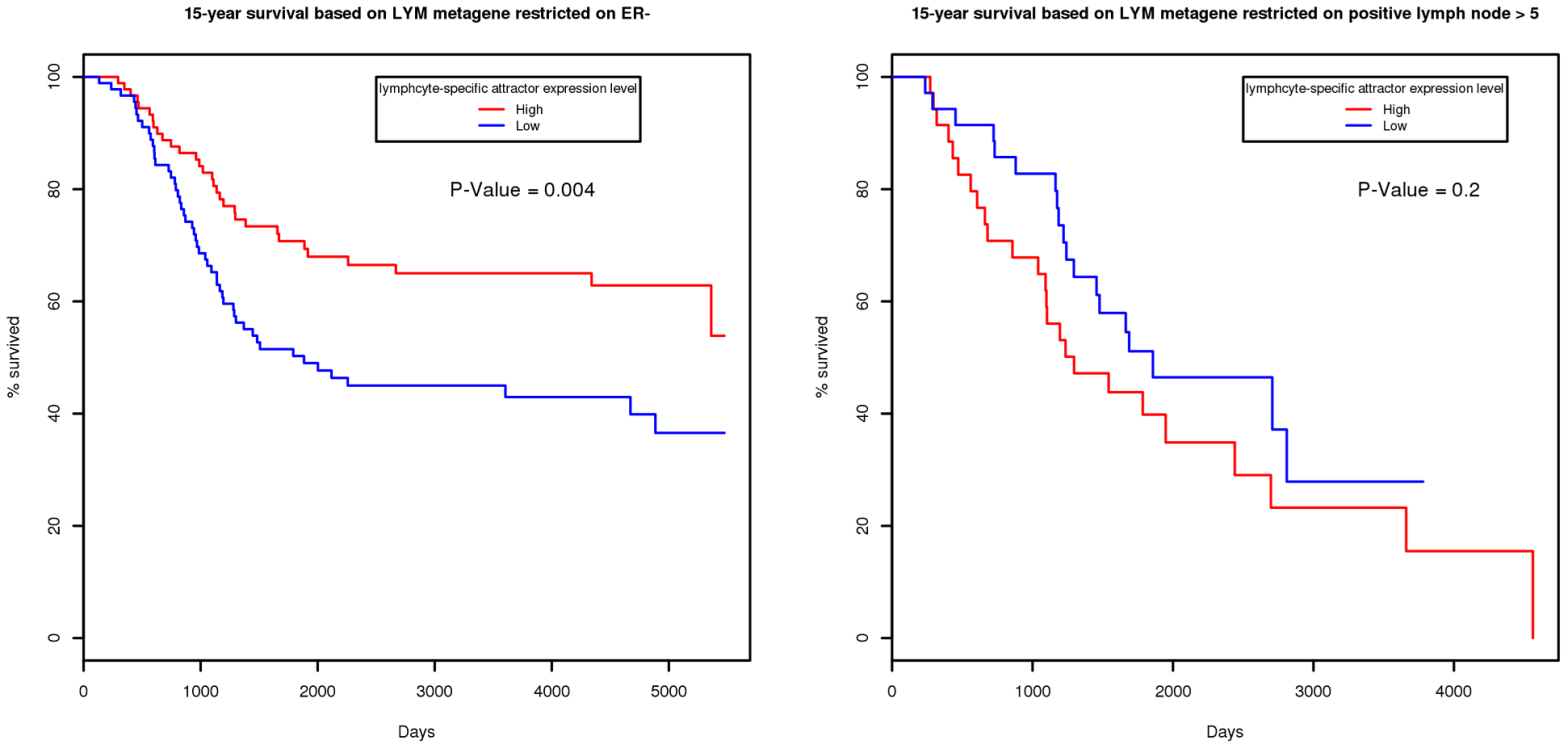
Kaplan-Meier curve of lymphocyte-specific attractor metagenes in breast cancer dataset. For ER-negative patients, the expression of the attractor is highly protective (high expression implies longer survival, left). However, when multiple lymph nodes are already affected, the expression of the attractor has a reversed effect on survival. When we restrict the samples to those with more than five positive lymph nodes, higher expression of the lymphocyte-specific attractor implies shorter survival (right), although the association is not significant due to the limited number of samples (76).

We found the attractor to be strongly protective in ER-negative breast cancers. As shown in Figure 4, the Kaplan-Meier 15-year survival curve restricted to ER-negative reveals association with longer survival (*P* = 0.004). Although the precise underlying biological mechanisms are unclear, it appears that this effect is due to some type of immune system recruitment to fight cancer. Interestingly, however, this effect is reversed in the presence of multiple positive lymph nodes. Indeed, the corresponding Kaplan-Meier curve shown on the right side of Figure 4 when restricted to patients with more than five positive lymph nodes demonstrates that the presence of the signature is associated with bad prognosis. This reversal may be explained by the fact that the presence of the lymphocyte-specific signature when lymph nodes are already affected implies that the cancer has obtained a (devastating for the patient) tolerance to this type of immune system recruitment.

### Other Attractors

#### Chr8q24.3 amplicon attractor

Amplification in chr8q24 is often considered to be associated with cancer because of the presence of the *MYC* (aka *c-Myc*) oncogene at location 8q24.21. Indeed, *MYC* is one of 157 genes in “amplicon 8q23-q24” previously identified [35] in an extensive study of the breast cancer “amplicome” derived from 191 samples.

We found, however, that the core of the amplified genes occurs at location 8q24.3 and this is, in fact, our most prominent multi-cancer amplicon attractor. Core genes of the attractor are *PUF60* (aka *FIR*), *EXOSC4*, *SHARPIN*, *HSF1*, *BOP1*, *SLC52A2*. It is known that PUF60 can repress c-Myc via its far upstream element (FUSE), although a particular isoform was found to have the opposite effect [36]. The other genes may also play important roles. For example, *HSF1* (heat shock transcription factor 1) has been associated with cancer in various ways [37]. It was found [38] that HSF1 can induce genomic instability through direct interaction with CDC20, a key gene of the mitotic CIN attractor mentioned above (listed in Table 2). Furthermore, HSF1 was found [39] required for the cell transformation and tumorigenesis induced by the ERBB2 (aka HER2) oncogene (see subsequent discussion of *HER2* amplicon) responsible for aggressive breast tumors.

The top ten genes of the chr8q24.3 attractor, ranked by the average of the highest five values of mutual information (Materials and Methods), are shown in Table 5. Interestingly, as shown in one of the attractors in Supplementary Table S1, an aneuploidy attractor involving a whole arm amplification of chr8q is also occasionally present in multiple cancer types, and this 8q whole arm amplification is the most prominent such aneuploidy attractor among all chromosomes.

**Table 5:** List of top ten genes in the chr8q24.3 and HER2 amplicons.

*chr8q24.3*			*HER2*		
Rank	Gene Symbol	Avg MI of Top 4 Datasets	Rank	Gene Symbol	Avg MI of Top 4 Datasets
1	EXOSC4	0.716	1	PGAP3	0.794
2	PUF60	0.659	2	ERBB2	0.793
3	BOP1	0.653	3	STARD3	0.768
4	SLC52A2	0.639	4	MIEN1	0.764
5	SHARPIN	0.634	5	GRB7	0.718
6	HSF1	0.616	6	PSMD3	0.602
7	FBXL6	0.615	7	GSDMB	0.539
8	CYC1	0.608	8	ORMDL3	0.498
9	SCRIB	0.552	9	MED24	0.414
10	GPAA1	0.551	10	MED1	0.400

Furthermore, prognostic associations involving the 8q24.3 amplicon have recently been recognized in various cancers [40], [41].

#### Chr17q12 HER2 amplicon attractor

This amplicon is prominent in breast cancer [42] and we also found it present in some samples of ovarian cancer, but not as much in colon cancer. So we initially used the four datasets of breast and ovarian cancer for deriving the attractor. We found that *ERBB2* (aka *HER2*), *STAR3*, *GRB7* and *PGAP3* were the top-ranked genes, consistent with their known presence in the amplicon. We also found that gene *MIEN1* (aka *C17orf37*) was very highly ranked in the two datasets in which its probe set was present. *MIEN1* has recently been identified as an important gene within the 17q12 amplicon in various cancers including prostate cancer [43]. Therefore, we augmented the choice of datasets to the following seven, of which *MIEN1* is included in five: breast GSE2034, breast GSE32646, breast GSE36771, breast TCGA, ovarian GSE9891, ovarian GSE26193, ovarian TCGA. Table 5 shows the top ten genes ranked by the average of the top five scores of mutual information (Materials and Methods) in the seven datasets for each gene. The results suggest that the above-mentioned five genes, including *MIEN1*, are consistently strongly co-expressed, and therefore are likely “driver” genes in the amplicon.

In addition to the narrow *HER2* amplicon, it is known that sometimes a large amplicon extends to more than a million bases containing both *HER2* as well as *TOP2A* (one of the genes of the mitotic CIN attractor) at 17q21 [44]. We have observed that *TOP2A* indeed appears among the top 50 genes in terms of its association with the attractor in breast cancer. *HER2*/*TOP2A* co-amplification has been linked with better clinical response to therapy.

#### Estrogen receptor breast cancer attractor

We found this attractor clearly present only in breast cancer, and therefore we derived it using six breast cancer datasets (GSE2034, GSE3494, GSE31448, GSE32646, GSE36771, breast TCGA). Table 6 shows the top 50 genes ranked by the average mutual information (Materials and Methods) in these datasets, revealing that genes *AGR3, CA12*, *AGR2*, *GATA3*, *FOXA1*, *MLPH* and *TBC1D9* are strongly co-expressed with the estrogen receptor *ESR1* in the attractor. However, this co-expression is not as uniform as in the other attractors. For example, the progesterone receptor PGR appear in the list, but in reality it can be underexpressed even if the estrogen receptor ESR1 is not.

**Table 6:** Top 50 genes of the estrogen receptor breast cancer attractor.

Rank	Gene Symbol	Avg MI	Rank	Gene Symbol	Avg MI
1	AGR3	0.847	26	ERBB4	0.393
2	CA12	0.616	27	AR	0.383
3	FOXA1	0.613	28	P4HTM	0.383
4	GATA3	0.585	29	SLC44A4	0.380
5	MLPH	0.580	30	KDM4B	0.375
6	AGR2	0.570	31	GFRA1	0.374
7	ESR1	0.543	32	MAPT	0.370
8	TBC1D9	0.540	33	MYB	0.364
9	XBP1	0.460	34	DACH1	0.359
10	ANXA9	0.456	35	SLC7A8	0.359
11	PRR15	0.452	36	MAGED2	0.358
12	SCUBE2	0.444	37	FBP1	0.357
13	FSIP1	0.438	38	SLC22A5	0.355
14	TFF3	0.429	39	CMBL	0.346
15	SPDEF	0.429	40	DYNLRB2	0.346
16	NAT1	0.428	41	C6orf211	0.342
17	ABAT	0.423	42	GREB1	0.342
18	CCDC170	0.422	43	SIDT1	0.338
19	DNALI1	0.418	44	TTC39A	0.330
20	DEGS2	0.415	45	FAM214A	0.326
21	DNAJC12	0.411	46	IL6ST	0.324
22	SLC39A6	0.406	47	CXXC5	0.323
23	CAPN8	0.399	48	ACADSB	0.323
24	TFF1	0.397	49	CELSR1	0.322
25	THSD4	0.395	50	CLSTN2	0.322

### Comparison with Other Unsupervised Algorithms

The scope of the algorithm identifying attractor metagenes is different from that of other unsupervised methods, which are usually aimed at identifying subtypes or mutually exclusive clusters. Nevertheless, it is interesting to find to what extent other algorithms can produce multiple cancer signatures each of which appears in nearly identical form across different types. We applied three widely used methods, k-means clustering, principal component analysis and hierarchical clustering on the six cancer datasets used in this paper. In all cases, we listed the top fifty genes in each cluster and applied the same clustering algorithm as in the main text to find common genes among them and group them together. The results are shown in Supplementary Text S2 and Supplementary Tables S5, S6, S7. We found that, in all cases, these well-established methods cannot identify multiple universal metagenes common in all six tested datasets.

### Using Attractor Metagenes as Proxies of Biomolecular Events

A biomolecular event, whether it is present in multiple cancer types or it is cancer specific, can be represented by a “consensus attractor metagene” after analyzing multiple datasets. To generate such consensus attractors, we use genes that were profiled by at least three of the six datasets, then rank individual genes in terms of their average mutual information (Materials and Methods) with the corresponding attractor metagenes across all datasets in which the gene was profiled.

**Figure 5:**
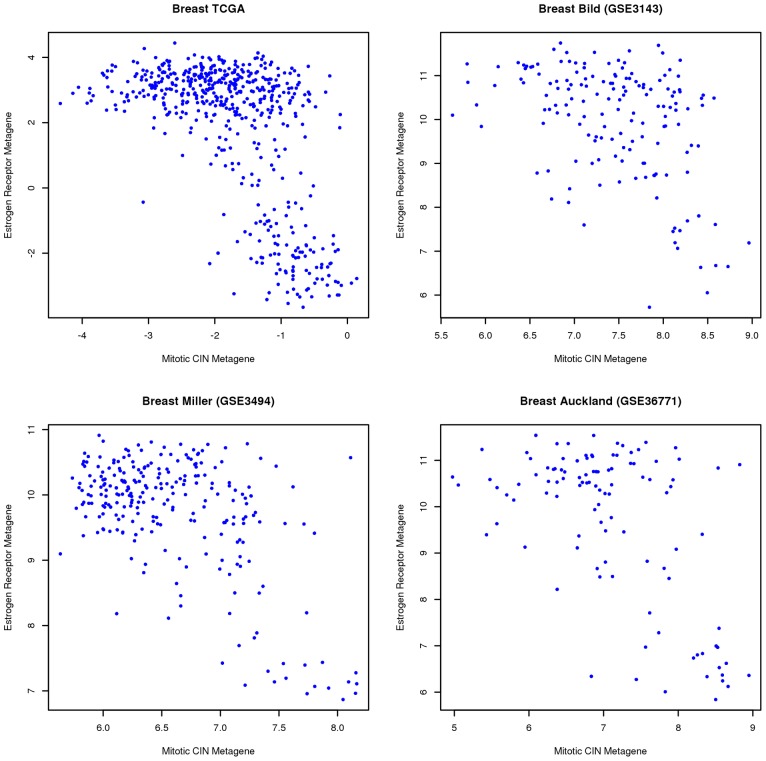
Scatter plots demonstrating the relationship between mitotic CIN attractor and estrogen receptor attractor in breast cancer. The two metagenes were defined to be “consensus attractors” after ranking individual genes in terms of their average mutual information with the corresponding attractor metagenes, across all datasets, and selecting the genes having average mutual information greater than 0.5. These criteria led to 59 genes in the consensus mitotic CIN attractor (the top 59 genes in Table 2), and *AGR3, ESR1*, *CA12*, *AGR2*, *GATA3*, *FOXA1*, *MLPH* and *TBC1D9* (the top eight genes in Table 6) in the consensus estrogen receptor breast cancer attractor. These scatter plots reveal that ER-negative breast tumors have high mitotic chromosomal instability, but not necessarily vice versa.

For example, Figure 5 contains scatter plots from four different rich breast cancer datasets connecting the mitotic CIN and estrogen receptor attractors. It has previously been reported [45] that breast tumors with high chromosomal instability are predominantly of the estrogen receptor negative phenotype. Although these scatter plots cannot be used for precise conclusions, they do suggest in all cases that ER-negative tumors have high mitotic chromosomal instability (or equivalently that low chromosomal instability implies that the tumor is ER-positive). The reverse relationship, however, is not as clear.

## Discussion

Gene expression analysis has resulted in several cancer types being further classified into subtypes labeled, e.g. as “mesenchymal” or “proliferative.” Such characterizations, however, may sometimes simply reflect the presence of the mesenchymal transition attractor or the mitotic chromosomal instability attractor, respectively, in some of the analyzed samples. Similar subtype characterizations across cancer types often share several common genes, but the consistency of these similarities has not been significantly high.

By contrast, using an unconstrained algorithm independent of subtype classification or dimensionality reduction, we identified several attractors exhibiting remarkable consistency across many cancer types, suggesting that each of them represents a precise biological phenomenon present in multiple cancers.

We found that the mesenchymal transition attractor is significantly present only in samples whose stage designation has exceeded a threshold, but not in all of such samples. On the other hand, the absence of the mesenchymal transition attractor in a profiled high-stage sample (or the absence of the mitotic chromosomal instability attractor in a profiled high-grade sample) does not necessarily mean that the attractor is not present in other locations of the same tumor. Indeed, it is increasingly appreciated [46] that tumors are highly heterogeneous. Therefore it is possible for the same tumor to contain components, in which, e.g., some are migratory having undergone mesenchymal transition, some other ones are highly proliferative, etc. If so, attempts for subtype classification based on one particular site in a sample may be confusing.

Existing molecular marker products make use of multigene assays that have been derived from phenotypic associations in particular cancer types. For breast cancer, biomarkers such as Oncotype DX [47] and Mammaprint [48] contain several genes highly ranked in our attractors. For example, most of the genes used for the Oncotype DX breast cancer recurrence score directly converge to one of our identified attractors: *MMP11* to the mesenchymal transition attractor; *MKI67* (aka *Ki-67*), *AURKA* (aka *STK15*), *BIRC5* (aka *Survivin*), *CCNB1*, and *MYBL2* to the mitotic CIN attractor; *CD68* to the lymphocyte-specific attractor; *ERBB2* and *GRB7* to the *HER2* amplicon attractor; and *ESR1*, *SCUBE2*, *PGR* to the estrogen receptor attractor.

We envision, instead, a multi-cancer biomarker product that will include detection of the level of expression of each of the key attractor metagenes. These levels would need to be combined in different ways in different cancer types, but each of the metagenes would indicate the same attribute and the contributions of each component will be cleanly distinguished. Even though molecular marker genes in some existing products are already separated into groups that are related to our attractor designation, any improvement in diagnostic, prognostic, or predictive accuracy resulting from better such group designation and better choice of genes in each group would be highly desirable. We hope that the identification of the attractors of cancer, as presented here, will be valuable in that regard.

## Materials and Methods

The full code of the attractor finding algorithm is publicly available in the Sage Bionetworks Synapse platform at https://synapse.sagebase.org/#Synapse:syn1446295. In addition, we provide a pseudo-code in Supplementary Text S3.

### General Attractor Finding Algorithm

We chose the association measure 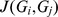 between genes to be a power function with exponent *a* of a normalized estimated information theoretic measure of the mutual information [49] 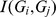 with minimum value 0 and maximum value 1 (see “Mutual information estimation” below; more sophisticated related association measures [50] can also be used, but computational complexity will be prohibitive). In other words, 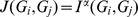, in which the exponent 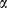
can be any nonnegative number. The value of 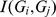 is set to zero if the Pearson correlation between the two genes is negative. Each iteration defines a new metagene in which the weight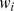 for gene 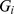 is equal to 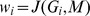 where 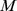 is the immediately preceding metagene. The process is repeated until the magnitude of the difference between two consecutive weight vectors is less than a threshold, which we chose to be equal to 10^−7^.

At one extreme, if 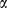 is sufficiently large then each of the seeds will create its own single-gene attractor because all other genes will always have near-zero weights. In that case, the total number of attractors will be equal to the number of genes. At the other extreme, if 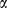 is zero then all weights will remain equal to each other representing the average of all genes, so there will only be one attractor. The higher the value of 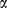, the “sharper” (more focused on its top gene) each attractor will be and the higher the total number of attractors will be. As the value of 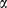 is gradually decreased, the attractor from a particular seed will transform itself, occasionally in a discontinuous manner, thus providing insight into potential related biological mechanisms.

We empirically found that an appropriate choice of 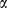 (in the sense of maximizing the strength of the attractor, as defined below) for general attractors is around 5, in which case there will typically be approximately 50 to 150 resulting attractors, each resulting from many attractee genes. An alternative to the power function can be a sigmoid function with varying steepness, but we found that the consistency of the resulting attractors was worse in that case.

As mentioned in the Introduction, an attractor metagene can also be interpreted as a set of the top genes of the attractor, i.e., a gene set that includes only the genes that are significantly associated with the attractor. One empirical choice for such a gene set would be to include only the genes whose mutual information (or the z-score thereof) with the attractor metagene exceeds a particular threshold. In fact, the attractor finding algorithm itself can be designed to discover “attractor gene sets,” without assigning weights to genes. In that case, metagenes are defined as simple averages of the genes in a gene set, and each iteration leads to a new gene set consisting of the new set of top-ranked genes in terms of their association with the previous metagene (gene set sizes can be constant or adaptively changing in various ways). We found, however, that such a method has the disadvantage of occasionally leading to attractors with significant overlap, which requires additional post-processing steps.

Identified attractors can be ranked in various ways. The “strength of an attractor” can be defined as the mutual information between the *n* ^th^ top gene of the attractor and the attractor metagene. Indeed, if this measure is high, this implies that at least the top *n* genes of the attractor are strongly co-expressed. We selected *n* = 10 as a reasonable choice, not too large, but sufficiently so to represent a real complex biological phenomenon of co-expression of at least ten genes. For amplicons, *n* = 5 is sufficient to ensure that the oncogenes are included in the co-expression). We use these choices when referring to the strength of an attractor.

The top genes of many among the found attractors are genomically localized. In that case the biomolecular event that they represent is often the presence of a particular copy number variation. In the cancer datasets that we tried, this phenomenon almost always corresponds to a local amplification event known as an amplicon. We therefore also devised a related amplicon-finding algorithm, custom-designed to identify localized amplicon-representing attractor metagenes, described below.

### Genomically Localized Attractor Finding Algorithm

To identify genomically localized attractors – almost always amplicons – we use the same algorithm but for each seed gene we restrict the set of candidate attractor genes to only include those in the local genomic neighbourhood of the gene, and we optimize the exponent a so that the strength of the attractor is maximized. Specifically, we sort the genes in each chromosome in terms of their genomic location and we only consider the genes within a window of size 51, i.e., with 25 genes on each side of the seed gene. We further optimize the choice of the exponent 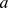 for each seed, by allowing 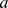 to range from 1.0 to 6.0 with step size of 0.5 and selecting the attractor with the highest strength.

Because the set of allowed genes is different for each seed, the attractors will be different from each other, but “neighbouring” attractors will usually be very similar to each other. Therefore, following exhaustive attractor finding from each seed gene in a chromosome, we apply a filtering algorithm to only select the highest-strength attractor in each local genomic region, as follows: For each attractor, we rank all the genes in terms of their mutual information with the corresponding attractor metagene and we define the range of the attractor to be the chromosomal range of its top 15 genes. If there is any other attractor with overlapping range and higher strength, then the former attractor is filtered out. This filtering is done in parallel, so elimination of attractors occurs simultaneously.

### Mutual Information Estimation

Assuming that the continuous expression levels of two genes 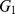 and 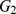 are governed by a joint probability density 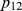 with corresponding marginal 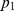 and 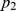, the mutual information 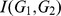 is defined as the expected value of 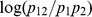. It is a non-negative quantity representing the information that each one of the variables provides about the other. The pairwise mutual information has successfully been used as a general measure of the correlation between two random variables. We compute mutual information with a spline-based estimator [51] using six bins in each dimension. This method divides the observation space into equally spaced bins and blurs the boundaries between the bins with spline basis functions using third-order B-splines. We further normalize the estimated mutual information by dividing by the maximum of the estimated 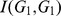 and 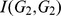, so the maximum possible value of 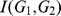 is 1.

### Pre-processing Gene Expression Datasets

We used Level 3 data when directly available, and imputed missing values using a k-nearest-neighbour algorithm with k = 10, as implemented in R [52]. We normalized the other datasets on the Affymetrix platform using the RMA algorithm as implemented in the *affy* package in Bioconductor [53]. To avoid biasing attractor convergence with multiple correlated probe sets of the same gene, we summarized the probe set-level expression values into the gene-level expression values by taking the mean of the expression values of probe sets for the same genes. We used the annotations for the probe sets given in the *jetset* package [54].

To investigate the associations between the attractor metagene expression and the tumor stage and grade, we used the following annotated gene expression datasets. For stage association: Breast (GSE3893), TCGA Ovarian, Colon (GSE14333). For grade association: Breast (GSE3494), TCGA Ovarian, Bladder (GSE13507). For Breast GSE3494 we used only the samples profiled by U133A arrays. For Breast GSE3893 we combined two platforms by taking the intersections of the probes in the U133A and the U133Plus 2.0 arrays. For datasets profiled by Affymetrix platforms all the datasets were normalized using the RMA algorithm. For Bladder GSE13507 normalization was done as provided in the GEO.

### *P* Value Evaluation

*P* values for gene set enrichment were evaluated with the cumulative hypergeometric distribution using the total number of genes in each dataset.

The significance of the consistency of the mesenchymal transition and mitotic CIN attractors was evaluated as follows: Supplementary Table S1 contains 210 gene sets from six cancer datasets. Each of the gene sets contains 50 genes. The mesenchymal transition metagene has eight genes (COL5A2, COL1A2, SPARC, CTSK, FBN1, VCAN, AEBP1, SERPINF1) common across all six datasets. The mitotic CIN metagene has 13 common genes (CENPA, DLGAP5, KIF2C, CCNB2, MELK, CCNA2, KIF20A, HJURP, NUSAP1, BUB1, TTK, KIF11, NCAPH) across all six datasets. To evaluate the significance of the consistency across the six datasets, we randomly generated 210 gene sets with the same sizes as those in the Table. In other words, we randomly selected 50 genes out of the 11,395 common genes to generate a random gene set. We created 210 such random gene sets, and then assigned them to six different datasets according to the settings in the Table. We then performed the clustering algorithm described in Materials and Methods. Each time, we counted the maximum number of genes common in all six datasets, and we repeated this process ten million times. This constitutes a conservative way of evaluating consistency, in the sense that for each random experiment we only record the maximum number of common genes in the gene set cluster, and we created random gene sets using only the common genes in the six datasets. In these ten million experiments, it never occurred that more than one gene was common in all six datasets. Therefore, the corresponding *P* value for both the mesenchymal transition metagene as well as the mitotic CIN metagene is less than 10^−7^, and is in fact much lower than that given the large number (8 and 13 respectively) of the common genes.

## Supporting Information

Table S1 General attractors identified from the six datasets.
(XLS)

Table S2 Genomically localized attractors identified from the six datasets.
(XLS)

Table S3 Association of mesenchymal transition attractor with tumor stage.
(XLS)

Table S4 Association of mitotic CIN attractor with tumor grade.
(XLS)

Table S5 Common clusters from the six datasets using k-means.
(XLS)

Table S6 Common clusters from the six datasets using principal component analysis.
(XLS)

Table S7 Common clusters from the six datasets using hierarchical clustering.
(XLS)

Text S1 Datasets and methods used to derive Supplementary Tables S1, S2, S3, S4.
(DOCX)

Text S2 Comparison with other unsupervised algorithms.
(DOCX)

Text S3 Pseudo-code for attractor metagene finding algorithm.
(DOCX)
