# Supplementary material for: Biomolecular Events in Cancer Revealed by Attractor Metagenes
Source: PLoS Comput Biol. 2013 Feb 21;9(2):e1002920. doi: 10.1371/journal.pcbi.1002920 (PMC3581797; doi:10.1371/journal.pcbi.1002920)
Supplement: Text S1 — Datasets and methods used to derive Supplementary Tables S1, S2, S3, S4. (DOCX) [file pcbi.1002920.s008.docx]

**Supplementary Text S1:** Datasets and methods used to derive

Supplementary Tables S1, S2, S3, S4.

**Datasets used to derive attractors in Supplementary Tables S1 and S2**

| **Dataset** | **Sample Size** | **Platform** |
| --- | --- | --- |
| Breast Wang (GSE2034) | 286 | Affymetrix HG-U133A |
| Breast TCGA | 536 | Agilent 244K Custom Gene Expression G4502A-07-3 |
| Colon Jorrison (GSE14333) | 290 | Affymetrix HG-U133Plus 2.0 |
| Colon TCGA | 154 | Agilent 244K Custom Gene Expression G4502A-07-3 |
| Ovarian Tothill (GSE9891) | 285 | Affymetrix HG-U133Plus 2.0 |
| Ovarian TCGA | 584 | Affymetrix HG-U133A |

**Supplementary Table S1** (General attractors identified from the six datasets) was created as follows: After applying the attractor finding algorithm in the six datasets of Supplementary Table 1, we filtered out any attractors that resulted from less than three attractee (seed) genes. To identify common attractors in different datasets, we first rank the genes in each attractor according to their mutual information with the attractor metagene, selecting the top 50 genes as its representative “attractor gene set.” We then perform hierarchical clustering on the attractor gene sets. The clustering algorithm iteratively defines “attractor clusters,” each of which only contains attractors from distinct datasets, so that its maximum size is six. We define the “similarity score” between two attractor clusters to be the number of overlapping genes among all possible pairs of attractor gene sets between two attractor clusters. If two attractor clusters both contain gene sets from the same datasets, then we do not cluster them together. Starting from the two attractor gene sets with highest similarity score, we proceed until there is no attractor cluster pair that can be further clustered together. Attractors are ranked in terms of their minimum strength among datasets in which they exist.

**Supplementary Table S2** (Genomically localized attractors identified from the six datasets) was created as follows: We rank all genomically localized attractors in each dataset according to their strength and perform the same clustering algorithm as described above, except that the attractor gene sets have size 15 and the similarity score is set to 1 if two attractors are overlapping and 0 if their ranges are disjoint. Attractors are ranked in terms of their minimum strength among datasets in which they exist.

**Supplementary Table S3** (Association of mesenchymal transition attractor with stage) contains fold change-ranked listings of differentially expressed genes in three cancer datasets from different types (breast GSE3893, ovarian TCGA and colon GSE14333) that were annotated with clinical staging information. Check marks indicate the genes that are among the top 100 genes of the attractor (listed in Table 1)

**Supplementary Table S4** (Association of mitotic CIN attractor with grade) contains fold change-ranked listings of differentially expressed genes in three cancer datasets from different types (breast GSE3494, ovarian TCGA and bladder GSE13507) that were annotated with clinical grade information. Check marks indicate the genes that are among the top 100 genes of the attractor (listed in Table 2)
