# Supplementary material for: Biomolecular Events in Cancer Revealed by Attractor Metagenes
Source: PLoS Comput Biol. 2013 Feb 21;9(2):e1002920. doi: 10.1371/journal.pcbi.1002920 (PMC3581797; doi:10.1371/journal.pcbi.1002920)

**Supplementary Text S2:** Comparison with other unsupervised algorithms

Using k-means clustering: We used the *kmeans* function provided in R. The number of clusters k is the one at which the reduction of within group sum of squares decreases most significantly, which gives the “elbow” of the curve. In the Figure at the next page of this Supplementary Text S2, we show the within group sum of squares as k increases, and mark the “elbow” of the curve where k is chosen. In Supplementary Table S5 we list the top 50 genes in each cluster as ranked by their correlation with the center. As shown in the Table, there are no identified common clusters with significant overlap in all datasets.

Using principal component analysis: We used the *prcomp* function provided in R. In Supplementary Table S6 we list the top 50 genes in terms of their absolute values of the loadings in the first ten principal components for each data set. As shown in the Table, the amount of significant overlap in all datasets is significantly smaller than that in the case of the attractor metagenes. Interestingly, however, the mesenchymal transition attractor in particular was clearly identified with prominent presence of gene *COL11A1*, as we had derived [10] using a supervised methodology based on tumor stage.

Using hierarchical clustering: We used the *hclust* function provided in R with the average linkage and the Pearson correlation coefficient as the similarity metric. We cut the tree generated by the algorithm at 200 clusters. With this choice, after eliminating small clusters that have less than ten genes, we get 50 to 100 remaining clusters in each dataset, which is approximately the same number of attractor metagenes that we got from each dataset. Other choices close to this number gave similar results. Supplementary Table S7 shows the top 50 genes ranked by their correlation with the metagene created by averaging the genes in the cluster. The amount of significant overlap is significantly smaller. Furthermore, the total number of clusters present in all datasets, even with reduced overlap, is much smaller as well.


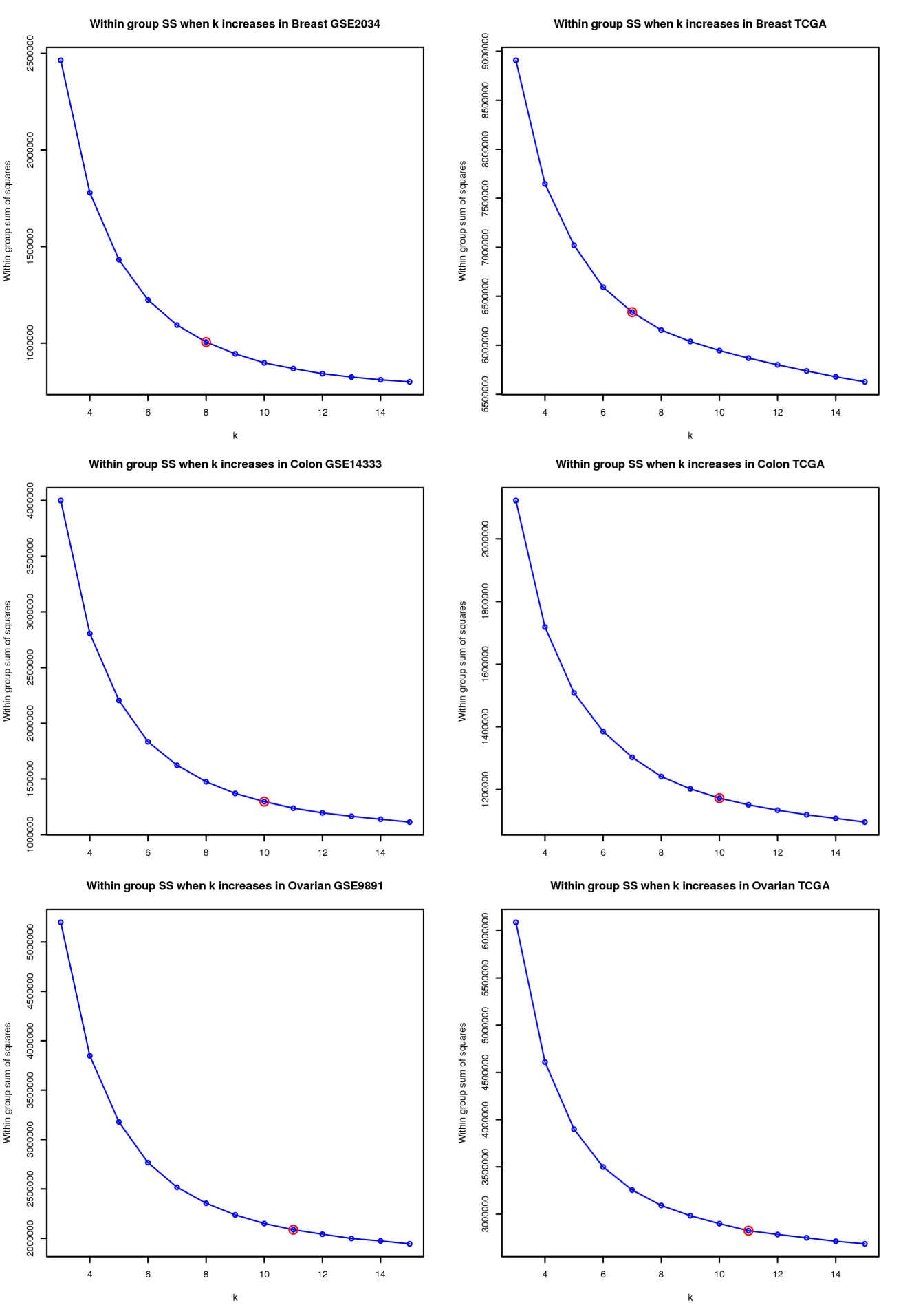

Supplement: Text S2 — Comparison with other unsupervised algorithms. (DOCX) [file pcbi.1002920.s009.docx]
