# Supplementary material for: Biomolecular Events in Cancer Revealed by Attractor Metagenes
Source: PLoS Comput Biol. 2013 Feb 21;9(2):e1002920. doi: 10.1371/journal.pcbi.1002920 (PMC3581797; doi:10.1371/journal.pcbi.1002920)
Supplement: Text S3 — Pseudo-code for attractor metagene finding algorithm. (DOCX) [file pcbi.1002920.s010.docx]

**Supplementary Text S3:** Pseudo-code of attractor metagene finding algorithm

1. Start from a seed gene $g_{seed}$.
2. Calculate the associations $I(g_{seed}; g_{i})$ between each gene $g_{i}$ and $g_{seed}$
3. Create a metagene $m_{0}$ using the weighted average of all the genes, with the weights $w_{i}$ being a function of $I(g_{seed};g_{i})$, i.e., $w_{i}=f(I(g_{seed};g_{i}))$
4. Given metagene $m_{0}$, calculate the associations $I(m_{0};g_{i})$ between metagene $m_{0}$ and each gene $g_{i}$
5. Create metagene $m_{1}$ using $w_{i}=f(I(m_{0};g_{i}))$
6. Repeat iteration until $m_{j+1}=m_{j}$, or until a preset maximum iteration number is reached.
7. The converged $m_{j}$ is the attractor metagene given by $g_{seed}$
